# Weak Coherence in Abundance Patterns Between Bacterial Classes and Their Constituent OTUs Along a Regulated River

**DOI:** 10.3389/fmicb.2015.01293

**Published:** 2015-11-26

**Authors:** Clara Ruiz-González, Guillem Salazar, Ramiro Logares, Lorenzo Proia, Josep M. Gasol, Sergi Sabater

**Affiliations:** ^1^Institute of Aquatic Ecology, University of GironaGirona, Spain; ^2^Departament de Biologia Marina i Oceanografia, Institut de Ciències del Mar (ICM-CSIC)Barcelona, Spain; ^3^Département des Sciences Biologiques, Université du Québec à MontréalMontréal, QC, Canada; ^4^Catalan Institute for Water Research, Scienfitic and Technological Parc of the University of GironaGirona, Spain

**Keywords:** freshwater bacterioplankton, bacterial community composition, high-rank lineages, ecological coherence, river gradient, 454-pyrosequencing

## Abstract

Deductions about the ecology of high taxonomic bacterial ranks (i.e., phylum, class, order) are often based on their abundance patterns, yet few studies have quantified how accurately variations in abundance of these bacterial groups represent the dynamics of the taxa within them. Using 454-pyrosequencing of the 16S rRNA gene, we investigated whether the changes in abundance of six dominant bacterial classes (Actinobacteria, Beta-/Alpha-/Gamma-proteobacteria, Flavobacteria, and Sphingobacteria) along a large dam-regulated river are reflected by those of their constituent Operational Taxonomic Units (OTUs; 97% similarity level). The environmental impact generated by the reservoirs promoted clear compositional shifts in all bacterial classes that resulted from changes in the abundance of individual OTUs rather than from the appearance of new taxa along the river. Abundance patterns at the class level represented the dynamics of only a small but variable proportion of their constituting OTUs, which were not necessarily the most abundant ones. Within most classes, we detected sub-groups of OTUs showing contrasting responses to reservoir-induced environmental changes. Overall, we show that the patterns observed at the class level fail to capture the dynamics of a significant fraction of their constituent members, calling for caution when the ecological attributes of high-ranks are to be interpreted.

## Introduction

Despite recent advances in our knowledge of bacterial communities associated to the development of high throughput sequencing technologies, it is still under debate whether bacterial high taxonomic ranks (i.e., phylum, class, order) form ecologically coherent units and to which extent their constituent taxa share functional traits and environmental preferences (e.g., Fierer et al., 2007; Philippot et al., 2010; Placella et al., 2012). Regardless of the level of resolution at which the taxonomic composition of bacteria is assessed, many studies end up collapsing diversity into broad phylogenetic levels for practical purposes, and interpretations about the ecology of these lineages are often based on the distribution patterns of these broad groups over environmental or spatio-temporal gradients. Thus, a question that remains open is how accurately variations in the abundances of major bacterial lineages reflect the ecology of most or just a fraction of their constituting taxa, and whether this pattern changes among major bacterial groups within a given ecosystem.

From an ecological point of view, a bacterial group (phylum or class) would behave as an ecologically coherent clade if most taxa within it respond similarly to changes in environmental conditions. More specifically, if a given high-rank group shows a decrease in abundance following a change in the environment, then such group could be regarded as an ecologically coherent entity if most of its constituting taxa also showed proportional reductions in their abundances without major changes in community composition. However, cases where changes in high-rank abundance are due to a complete substitution of the existing taxa by other better adapted to the new conditions, or due to a large increase in the relative abundances of previously rare taxa, would imply the presence of ecologically different sub-clusters within that high-rank group. The latter scenarios contradict the idea of functionally coherent units, and could lead to misleading interpretations of the ecological attributes of a particular lineage depending on the fraction of the constituent taxa that actually holds such attributes.

Considerable experimental and empirical evidence has led to the idea that high taxonomic ranks can show a certain degree of ecological coherence, despite the enormous phylogenetic, and physiological diversity that they harbor. For example, studies have reported recurrent seasonal or spatial patterns in the abundances of deeply branching bacterial lineages, such as phyla or classes (Gilbert et al., 2009; Ghiglione and Murray, 2012; Ruiz-González et al., 2013a; Staley et al., 2015) or clear changes in the abundance and distribution of high-rank bacterial phylotypes in response to environmental gradients or experimental manipulations (Bouvier and del Giorgio, 2002; Perez and Sommaruga, 2006; Ferrera et al., 2011; Barberán and Casamayor, 2012; Ren et al., 2015), suggesting that most dominant taxa within them share ecological preferences. Similarly, differences at these taxonomic levels have been reported in important ecological attributes such as the ability to use certain organic compounds (Cottrell and Kirchman, 2000; Vila-Costa et al., 2007; Alonso-Sáez et al., 2012; Sarmento and Gasol, 2012), the sensitivity to sunlight (Ruiz-González et al., 2013b) or resuscitation strategies after rewetting of dry soil (Placella et al., 2012). A hitherto unresolved question is to which extent the high-rank lineages' responses reported in many of these studies reflect the dynamics of most or just a small fraction of their constituent taxa.

Those studies that have specifically explored the taxonomic structure within high-rank bacterial groups challenge the ecological coherence at these levels and provide insight into their complex nature, even within single ecosystems. For example, Schauer et al. (2003) and Díez-Vives et al. (2014) found large seasonal changes in the composition of marine bacterial classes or phyla. Furthermore, different subgroups within typical high-rank bacteria were found to respond differently to the same environmental variables (Lindström et al., 2005; Newton et al., 2007; Ren et al., 2015) or to display distinct functional profiles (Teeling et al., 2012) or substrate uptake capabilities (Salcher et al., 2013). This evidences that even abundant taxa within a certain lineage can differ largely in their ecological preferences. In addition, the fact that different phyla or classes are known to harbor very different taxonomic or phylogenetic diversity levels (e.g., Newton et al., 2011) implies that different high-rank lineages will likely encompass variable degrees of ecological coherence within them, as has been shown for the human microbiota (Koeppel and Wu, 2012). This means that the abundance patterns of different bacterial lineages will likely provide a variable insight into the ecology of their constituent operational taxonomic units (OTUs), but this that has not yet been explicitly assessed in natural bacterial communities. Establishing the degree of ecological coherence within high bacterial ranks from particular ecosystems might allow determining the accuracy and generality of the conclusions derived from studies targeting major bacterial groups, and might eventually help to organize bacterial diversity into a treatable number of ecologically meaningful units.

In this context, our objective was to describe the compositional responses of different bacterial classes along a river interrupted by dams in order to explore which fraction of the OTU richness within each bacterial class is actually represented by the dynamics at the class level. The Ebro River (NE Spain) provides an ideal environment to examine the compositional succession within bacterial high-rank groups, since the presence of large reservoirs in the mid-lower reach creates pronounced changes in environmental conditions, which cause large and contrasting shifts in the relative abundance of dominant bacterial classes and phyla along the river (Ruiz-González et al., 2013a). We evaluated the degree of ecological coherence of the six most abundant bacterial classes in this system by 1) exploring changes in alpha- and beta-diversity in each class; 2) determining which fraction of the OTUs (operational taxonomic units, ≥97% similarity) within them showed the same patterns in abundance as its harboring class; and 3) identifying the existence of sub-groups of taxa with different ecological dynamics within each class. We are aware that OTUs have limitations as a unit of diversity since taxa delineated at the 97% (and even 99 or 100%) 16S rRNA gene sequence identity can contain ecologically distinct organisms (Staley et al., 2006; Ward et al., 2006; Koeppel and Wu, 2013). However, the 97% identity threshold is still the most widely used delineator of bacterial taxonomic units in microbial ecology studies (Logue et al., 2012; Shade et al., 2013; Lindh et al., 2015), including those addressing the ecological coherence of lineages (Koeppel and Wu, 2012), and thus we wanted to explore the coherence between bacterial classes and OTUs delineated at this specific similarity level. We acknowledge, though, that the units so defined may to a certain degree encompass ecologically different organisms.

## Materials and methods

### Study area, sampling, and contextual environmental parameters

The study system (Ebro River, NE Spain) and the sampling design have been previously described in Ruiz-González et al. (2013a). Briefly, it is a large (950 km long) river highly regulated by multiple reservoirs along its entire course. In particular, three large impoundments located in the mid-lower part cause abrupt changes in hydrologic and physico-chemical variables, leading to the establishment of compositionally different phytoplankton, and bacterial assemblages between up- and downstream waters (Sabater et al., 2008; Ruiz-González et al., 2013a).

Three samplings were carried out during mid and late summer (July, September) and in winter (December) 2011, each conducted over a 3-day period. Surface water samples were taken at six sites upstream and five downstream of the reservoir system, as well as one site located at the reservoir, covering a 330 km transect (see map in Ruiz-González et al., 2013a). Discharge was low and did not change largely between the three sampling campaigns, being consistently higher in downstream stations (range 130–160 m^3^ s^−1^) than in upstream (34–64 m^3^ s^−1^) or reservoir (51–78 m^3^ s^−1^) sites. At each station, water temperature, conductivity, pH, and dissolved oxygen were determined *in situ* with probes, and triplicate samples were filtered and stored until analysis for soluble reactive phosphorus, dissolved organic carbon, dissolved inorganic nitrogen, suspended solids, and chlorophyll-*a* concentrations. These values and methodological details are presented in Table 1 and the Methods section of Ruiz-González et al. (2013a).

**Table 1:** **ANOSIM ***R*** values between river reaches or seasons for the six studied bacterial classes**.

	**ANOSIM ***R*** values**	
	**Upstream vs. Downstream**	**Sampling campaign**
Actinobacteria	0.93^**^	0.22^*^
Betaproteobacteria	0.58^**^	0.29^*^
Alphaproteobacteria	0.86^**^	0.15^*^
Gammaproteobacteria	0.11^*^	0.09^*^
Flavobacteria	0.21^**^	0.15^*^
Sphingobacteria	0.49^**^	ns

All R for Bray-Curtis distance matrices values were calculated using 9999 permutations. Significance codes for R values: ^**^p < 0.0001, ^*^p < 0.001, ns = not significant.

### 454 Pyrosequencing and sequence processing

Due to the abundance of suspended matter, water samples were prefiltered through 1.2 μm filters (RAWG, Millipore) and 1–2.5 L were filtered onto 0.22 μm pore size filters (GSWP, Millipore) to obtain the free-living bacterial assemblage. Genomic DNA was extracted from the 0.22 μm filters with the Power Soil DNA extraction kit (MoBio, Solana Beach, CA, USA) following the manufacturer's protocol. The bacterial 16S rRNA gene V3/V4 region was amplified with the primers 341F (5′-CCTACGGGAGGCAGCAG-3′) and 907R (5′- CCGTCAATTCMTTTGAGTTT-3′, Muyzer et al., 1993, 1998). The PCR reactions followed an initial denaturation at 95°C for 5 min, 35 cycles (95°C, 30 s; 54°C, 40 s; 72°C, 1 min) and a final extension at 72°C for 10 min. Amplicons were pooled in equimolar concentrations into a single composite sample that was sequenced on a Roche 454 Life Sciences sequencer (Research and Testing Laboratories, Texas, USA).

Sequences between 150 and 600 bp (average 435 bp) were checked for quality [sliding window Phred average (50 bp) > 25] and denoised with DeNoiser (v 0.851, Reeder and Knight, 2010). Chimeras were detected with ChimeraSlayer (Haas et al., 2011) and removed, as well as singletons, and archaeal and chloroplast sequences.

Pyrosequencing data were processed using QIIME (Caporaso et al., 2010). Briefly, quality-checked sequences were clustered into operational taxonomic units (OTUs, ≥97% similarity) using UCLUST v1.2.22 q (Edgar, 2010). A representative sequence of each OTU was chosen and classified using the Ribosomal Database Project and SILVA taxonomies. Representative sequences were then aligned against the SILVA v108 reference alignment, and a phylogenetic tree was constructed using maximum likelihood in RAxML v. 7.2.8 (Stamatakis, 2006). Sequence data are available in the European Nucleotide Archive (ENA) database under the accession number PRJEB11464.

To enable comparisons between samples for beta-diversity analyses, the OTU table was randomly subsampled to ensure an equal number of sequences in each sample, based upon the sample with the least number of sequences (1000 sequences). Otherwise the non-rarefied OTU table was used (average 2740 sequences/sample, range 1000–6222 seqs). Although this number of sequences likely comprises the most abundant bacteria, we defined two abundance categories for exploratory purposes, “moderately abundant” (<0.1% of the pooled sequences) and “dominant” (>0.1% of the pooled sequences). A total of 32 samples were analyzed, since four winter samples provided too few sequences.

### Statistical analyses

The Shannon index was calculated as an estimate of bacterial taxonomic diversity, and the mean phylogenetic distance (MPD) was used to analyze changes in phylogenetic relatedness (Webb et al., 2002). The MPD estimates the mean phylogenetic pairwise distance (i.e., branch length) among groups of species within communities, and was calculated with the R package PICANTE (Kembel et al., 2010). Higher MPD values indicate more phylogenetically different taxa within a community. Both indices were calculated for all bacteria, separately for the six most abundant classes (i.e., Actinobacteria -phylum Actinobacteria-, Flavobacteria, and Sphingobacteria–Bacteroidetes- and Beta-, Alpha-, and Gammaproteobacteria–Proteobacteria-), and also for the different sub-groups of taxa within each class that were identified during our analyses (see Results). Significant (*p* < 0.05) differences in taxonomic diversity or phylogenetic relatedness among categories were analyzed through One-way analysis of variance (ANOVA).

Bray-Curtis or phylogeny-based UniFrac (Lozupone and Knight, 2005) beta diversity indices were calculated. This UniFrac distance matrix was constructed online from the phylogenetic tree using FastUnifrac (http://archive.is/http://bmf.colorado.edu/fastunifrac/). The two distances were visualized by nonmetric multidimensional scaling (NMDS) analyses done in *R*. The differences in beta diversity between upstream or downstream communities or among sampling campaigns were tested using ANOSIM (Analysis Of SIMilarity; Clarke, 1993) calculated for Bray-Curtis distance matrices performing 9999 permutations.

Correlations between different variables were calculated using the Pearson's correlation coefficient. Adjustments for multiple comparisons were applied using the Bonferroni correction procedure. A principal component analysis was performed with all the measured environmental variables (R package VEGAN, Figure S4) for exploring the responses of the OTUs within each class to the environmental gradients summarized by the two first principal components, and to compare these responses to those observed at the class level. In order to facilitate the interpretation of the PC axes, the PCA was rotated using the function “principal” from the R package pysch with the option “oblimin.” This resulted in a minimum loss of the variance explained (77 vs. 79% in the original PCA), and the two axes could be associated to seasonal (PC1) and spatial (PC2) variation in environmental conditions. All statistical analyses were performed with the JMP (v 9.0.1. SAS Institute) or R 3.0.0 (R Core Team, 2013) softwares.

## Results

The non-rarefied OTU table contained 95,927 quality-checked sequences with an average length of 435 bp that clustered into 957 OTUs (at 97% sequence similarity). After the rarefaction, 31,930 quality-checked sequences remained that clustered into 832 OTUs. Altogether, 15 bacterial phyla were detected, yet bacterial assemblages were mainly dominated by the phyla Actinobacteria (68% of the total number of classified sequences), followed by Proteobacteria (22%), and Bacteroidetes (7%). The former included exclusively the class Actinobacteria, while among Proteobacteria, the class Betaproteobacteria comprised 15% of the total sequences, Alphaproteobacteria 6%, and Gammaproteobacteria 0.6%. Finally, sequences belonging to the classes Flavobacteria and Sphingobacteria comprised most of Bacteroidetes sequences, and accounted for 4 and 3% of total sequences, respectively. Twelve other rarer phyla (<0.6% of all sequences) were also identified (details not shown). The OTU accumulation curve showed a clear plateau, suggesting that we had good coverage of the bacterial richness of the system (Figure S1).

### Shifts in class abundance, diversity, and composition along the river gradient

**Figure 1:**
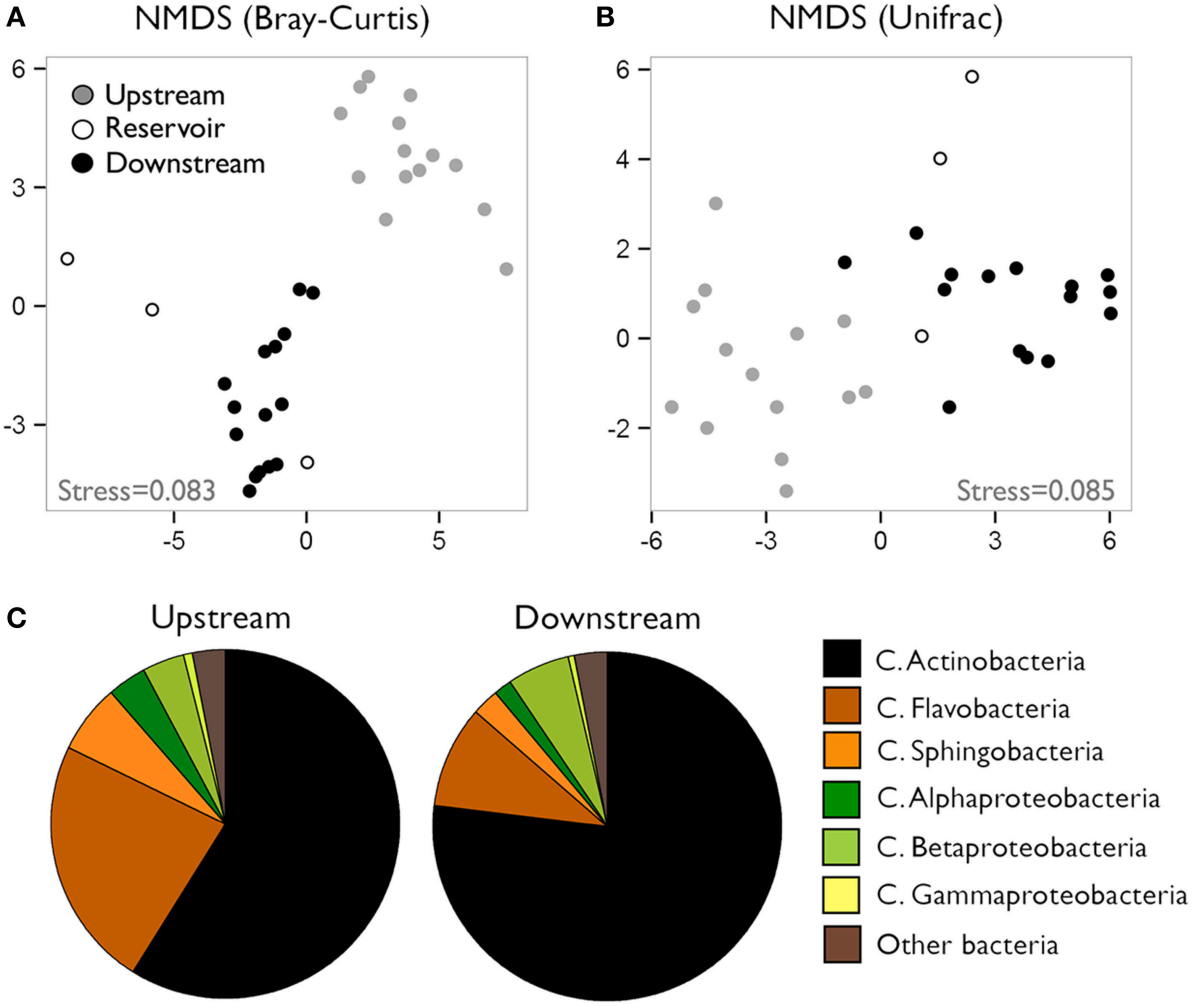
**(A)** Non-metric multidimensional scaling (NMDS) charts of community composition in the different river sections based on Bray-Curtis **(A)** and Weighted-Normalized Unifrac **(B)** distances. **(C)** Contribution of the different bacterial classes to community composition of upstream and downstream waters. Data presented as percent contribution of each group to total sequences (average of the three samplings).

**Figure 2:**
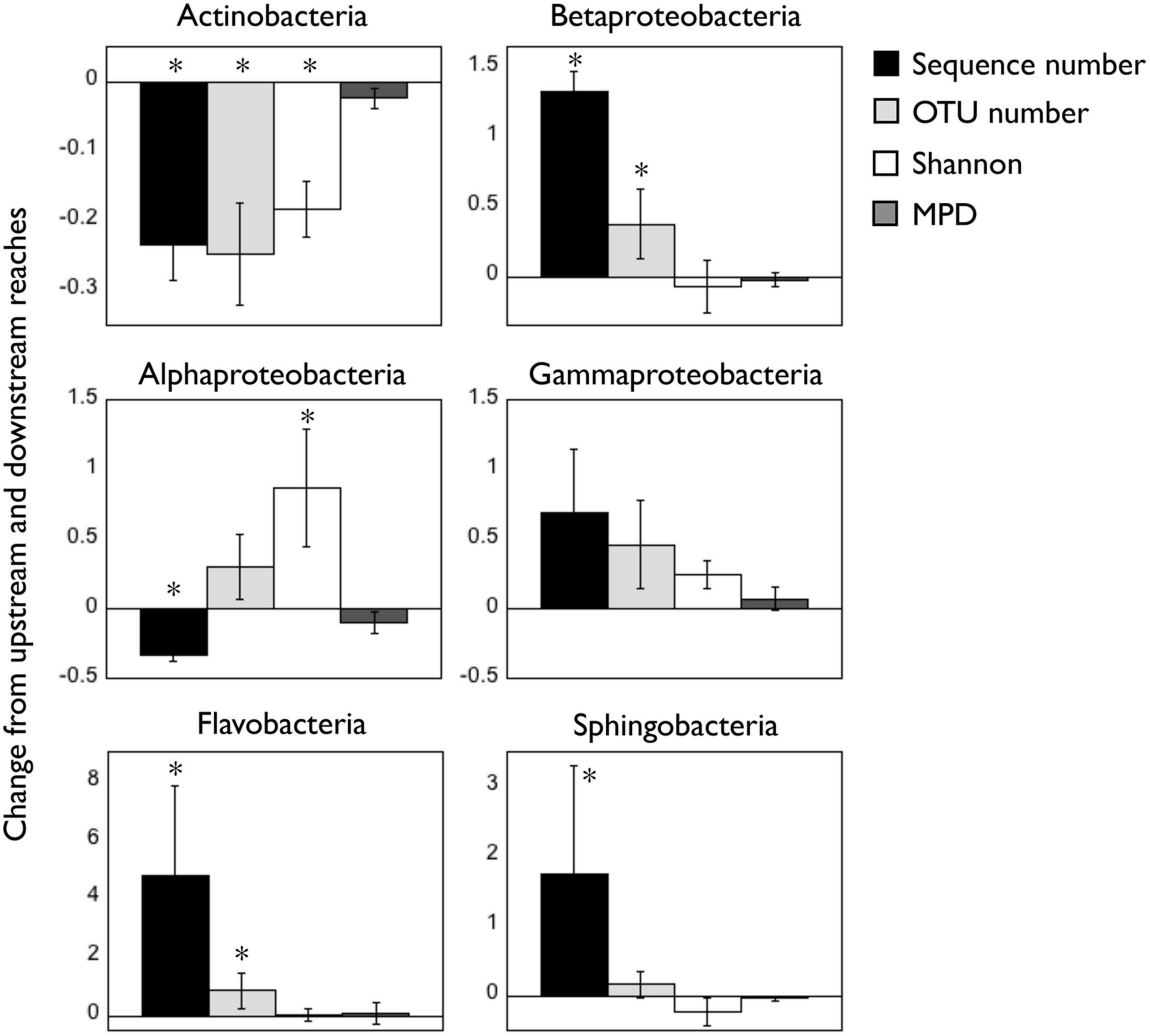
**Change in number of sequences, OTU richness, taxonomic diversity [Shannon], and mean phylogenetic distance [MPD] for the six bacterial classes between up- and downstream reaches**. Positive numbers indicate higher average values in upstream waters compared to downstream sites (ratio upstream/downstream), and the opposite is true for negative values. Asterisks indicate significant (*p* < 0.05) differences in those variables between both reaches. Note the different scales in Y axes. All values were calculated from the rarefied OTU table (1000 sequences/sample, see Materials and Methods) pooling the three samplings together.

We characterized the composition of bacterial communities from six sites upstream the reservoir, one site at the reservoir site, and five sites downstream the dam in three occasions. Communities from upstream sites differed largely from reservoir and downstream assemblages (Figures 1A,B, ANOSIM *R* = 0.93, *p* < 0.0001) regardless of the season, whereas the temporal variability was remarkably smaller (ANOSIM *R* = 0.22, *p* < 0.005). These clear spatial patterns were most likely caused by the large changes in environmental conditions associated to the presence of reservoirs, which resulted in abrupt decreases in conductivity, DIN and chlorophyll *a* concentrations and suspended particles from upstream to downstream reaches (for details see Ruiz-González et al., 2013a). In accordance to our previous results on the abundances of these bacterial classes assessed as CARD-FISH microscopic counts (Ruiz-González et al., 2013a), the sequence data also revealed clear abundance shifts between upstream and downstream waters for most bacterial classes (Figure 2), and both datasets showed remarkably similar patterns for all groups except for Gammaproteobacteria, likely due to the low sequence number recovered for this class (Figure S2). The differences in the percentage values between both approaches were likely due to the fact that while CARD-FISH targeted the whole bacterial assemblage, pyrosequencing was performed on the free-living bacterial fraction. However, the similar trends observed between sequence- and CARD-FISH-based relative abundances support that sequence patterns mirror the abundance dynamics at the class level. Overall, although Actinobacterial sequences dominated both upstream and downstream assemblages, a higher proportion of Actinobacteria and Alphaproteobacteria characterized downstream sites compared to upstream waters. In contrast, the rest of the groups tended to be more abundant in upstream sites (Figures 1C, 2).

Besides these shifts in abundance, the presence of reservoirs also led to variations in the alpha diversity of the six bacterial classes, suggesting that not all the taxa within these classes were responding equally to the environmental gradient. Changes in the number of sequences were sometimes accompanied by variations in OTU richness and/or the Shannon index of taxonomic diversity (Figure 2). In general most groups tended to harbor more OTUs where they were more abundant, whereas in the case of Alphaproteobacteria the increase in abundance was associated to a decrease in OTU richness and Shannon index (Figure 2). Betaproteobacteria, Gammaproteobacteria, Flavobacteria, and Sphingobacteria did not show clear patterns in the Shannon diversity index between upstream and downstream sites, and we did not find significant differences in the phylogenetic structure (MPD) of upstream and downstream populations. A comparison of the absolute values of these indices for the six bacterial classes is presented in Figure S3.

As these patterns suggested, we found significant differences in composition between upstream and downstream assemblages for all groups (Table 1), with Actinobacteria and Alphaproteobacteria, the two lineages that increased in downstream sites, showing the largest spatial differences. Likewise for all bacteria (Figures 1A,B), seasonal differences were always smaller than the spatial variability (Table 1), and thus we considered the three samplings together for all subsequent analyses.

### Compositional shifts: onset of new taxa vs. changes in relative abundances

**Figure 3:**
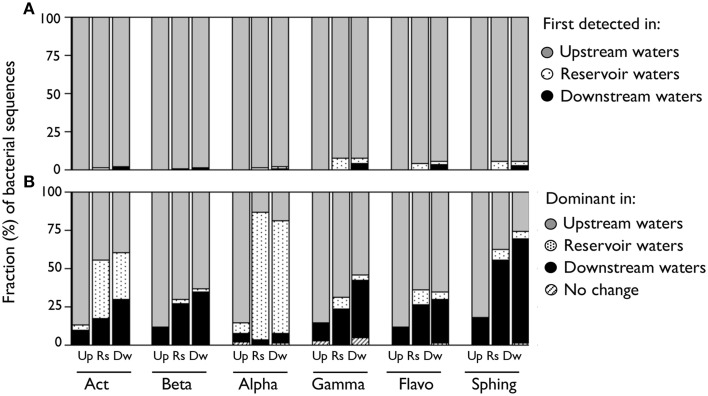
**(A)** Proportion of DNA sequences from each bacterial class associated to OTUs categorized by the farthest upstream environment where they were first detected considering a unidirectional flow of water from upstream to downstream. **(B)** Proportion of DNA sequences from each bacterial class associated to taxa categorized by the environment where they were more abundant. Those OTUs that had the same abundances in either reach were categorized as “No change.” Values are averages of the three samplings and are expressed as a fraction of the total sequences for each reach ([Up] Upstream; [Rs] Reservoir; [Dw] Downstream). This analysis was performed considering the non-rarefied OTU table. [Act] Actinobacteria; [Beta] Betaproteobacteria; [Alpha] Alphaproteobacteria; [Gamma] Gammaproteobacteria; [Flavo] C. Flavobacteria; [Sphing] Sphingobacteria.

We evaluated whether the observed changes in the composition of each class were due to the appearance of species along the riverine continuum, as opposed to variations in the relative abundances of previously existing taxa. To do so, we characterized each taxon depending on its origin or the environment where it was dominant. For assignation of the origin, we assigned each OTU to the farthest upstream river reach where it was first detected, assuming a unidirectional flow of water from upstream to downstream (see Crump et al., 2012). For assignation based on their dominance, we characterized each taxon depending on where they were most abundant (e.g., “upstream-dominant” OTUs were those whose summed sequence abundances from upstream waters where greater than those from reservoir and from downstream waters). We used the non-rarefied OTU table for these analyses, and for simplification, the results are presented as averages of the three samplings and of all the sites belonging to each of these river reaches. By these means, we found that most of the sequences found in reservoir or downstream waters belonged to OTUs that were already present in upstream waters (Figure 3A), suggesting that the onset of newly detected taxa did not seem to be responsible for the shifts in class composition along the river. Instead, when we categorized each OTU depending on the river reach where it was most abundant, we observed that the different composition of upstream and downstream class populations seemed to be due to changes in the relative abundances of ubiquitous taxa, although the patterns were more variable among bacterial groups (Figure 3B). We observed clear shifts in the contribution of these three groups (i.e., upstream-, reservoir-, and downstream-dominating groups) to the total sequences within upstream, reservoir, and downstream communities (Figure 3B). We detected the presence of reservoir-dominant taxa within all classes, yet only for Actinobacteria and Alphaproteobacteria these OTUs comprised a significant fraction of the total sequences in reservoir or downstream waters. In contrast, Beta-, Gammaproteobacteria, and Flavobacteria still harbored a remarkable proportion of upstream-dominant taxa in those sites located after the reservoir, while most Sphingobacterial sequences in reservoir or downstream assemblages belonged to downstream-dominant OTUs (Figure 3B).

### Coherence between abundance patterns of bacterial classes and their constituent OTUs

**Figure 4:**
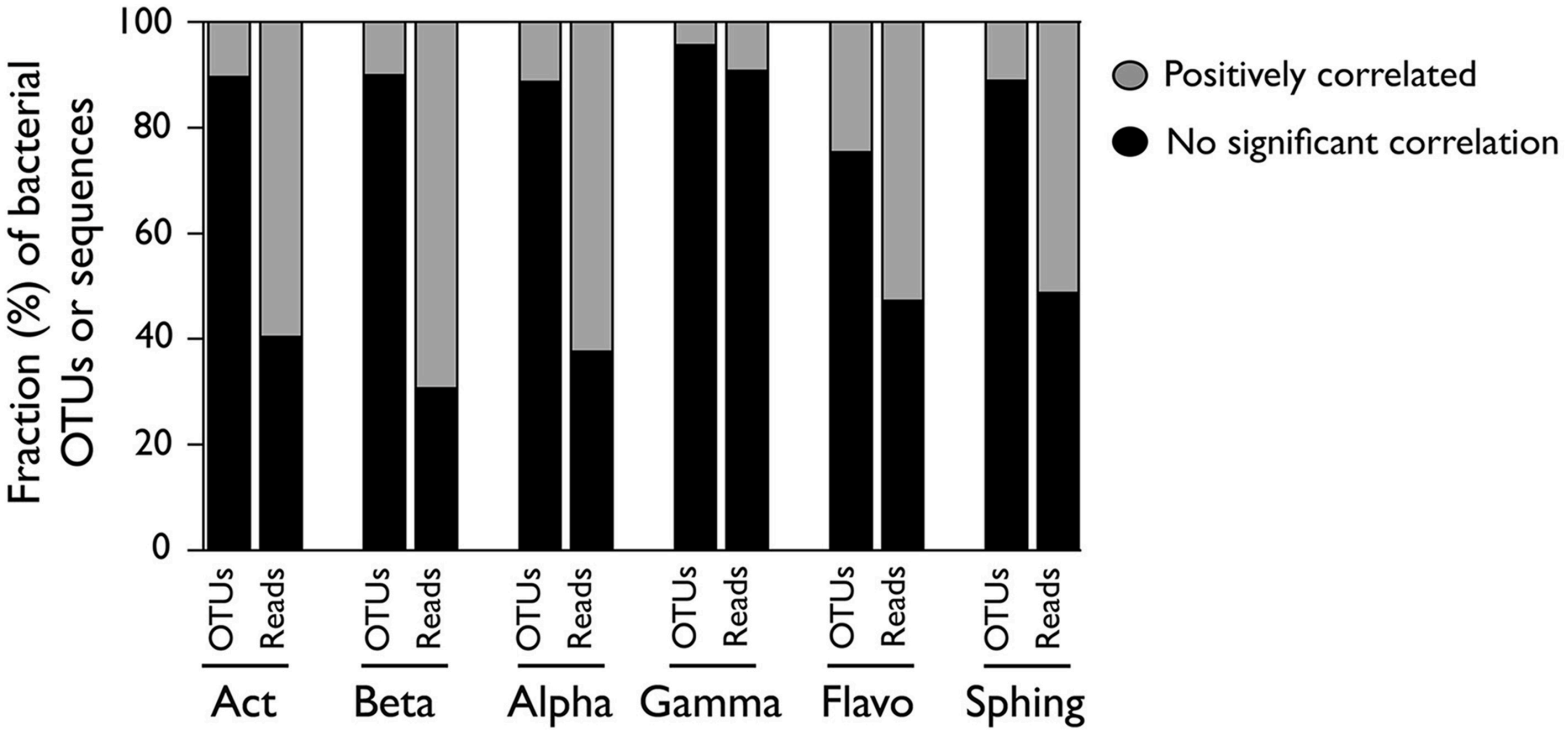
**OTUs within each bacterial class whose abundances were positively or not significantly correlated with the abundance of sequences in their parent class**. For each class, the proportion of OTUs (left bar) and the proportion of sequences represented by each of those OTUs (right bar) are presented. Correlations were performed by considering the non-rarefied OTU table and pooling the three samplings together (*n* = 34), and only Bonferroni-corrected significant (*p* < 0.05) correlations were considered. [Act] Actinobacteria; [Beta] Betaproteobacteria; [Alpha] Alphaproteobacteria; [Gamma] Gammaproteobacteria; [Flavo] **(C)**. Flavobacteria; [Sphing] Sphingobacteria.

To assess to what extent the patterns observed at the class level represent the dynamics of the OTUs within them, we compared the abundance patterns of each class to those of their constituent OTUs, and calculated the proportion of OTUs within each class that showed either positive, negative, or not significant correlations with the abundance patterns at the class level (Figure 4). We observed that for most classes, a very small fraction of the constituting OTUs showed positive correlations with class abundance, with values that ranged from 4% in Gammaproteobacteria to 25% in Flavobacteria. In terms of their contribution to total sequences, though, these OTUs comprised a larger proportion of the class, ranging from 9% (Gammaproteobacteria) up to 70% (Betaproteobacteria) of the retrieved sequences.

**Figure 5:**
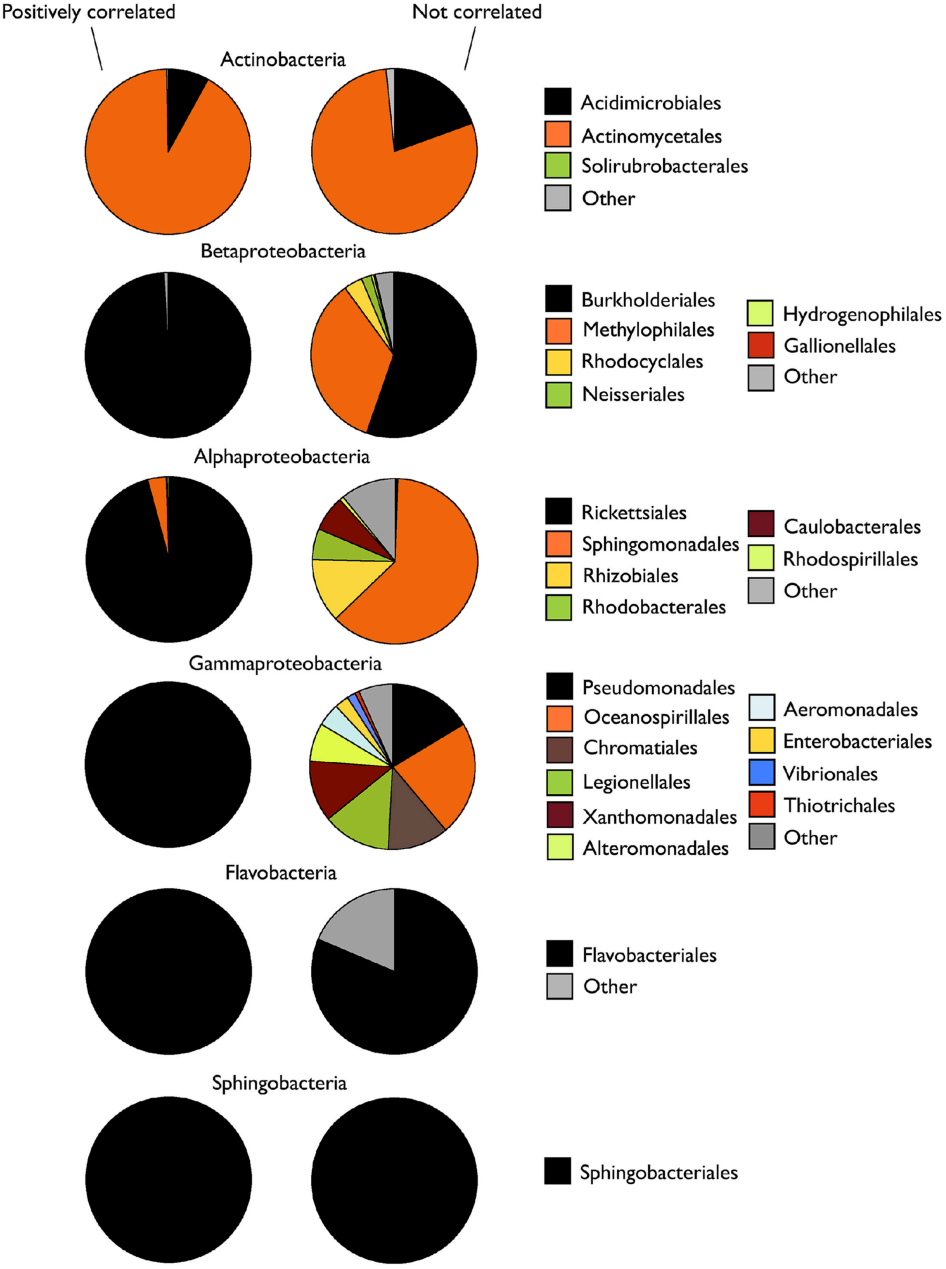
**Taxonomic composition of the OTUs within each bacterial class whose abundances were positively or not significantly correlated with the abundance of sequences of their parent class**. Data are presented as percent contribution of each taxonomic order to total sequences (average of the three samplings).

In all cases, the group of taxa that drove the dynamics of the class harbored significantly lower taxonomic diversity (Shannon index) than the non-correlating taxa (Table 2). Accordingly, MPD was also lower among positively-correlating OTUs, although in the case of Alpha- and Gammaproteobacteria these differences were not significant. The analysis of the taxonomic composition of these two groups within each class showed that, while the positively-correlated OTUs belonged to one or two taxonomic orders, the non-correlated ones were clearly more phylogenetically diverse, at least for the three proteobacterial classes (Alpha-, Beta-, Gamma-; Figure 5). Remarkably, although we expected to find most of the dominant OTUs (>0.1% of the pooled sequences) within those responsible for the patterns at the class level, in most cases the non-correlating OTUs contained a higher proportion of the “dominant” taxa within each class (Table 2). Likely due to the low number of sequences recovered, no Gammaproteobacterial OTU was categorized as “dominant.”

**Table 2:** **Comparison among different features associated to the groups of taxa whose abundances were positively or not significantly correlated [No correl] with the number of sequences of their parent class: number of sequences, OTUs, proportion (%) of the “dominant” OTUs [>0.1% of the pooled sequences] within each class contained by each group, average taxonomic diversity (Shannon Index), and mean phylogenetic distance [MPD]**.

		**Sequence number**	**Number of OTUs**	**% “dominant”**	**Diversity**	**MPD**
Actinobacteria	Positive	39163	40	36	**1.5**	**0.39**
	No correl	26601	347	64	**2.6**	**0.60**
Betaproteobacteria	Positive	9435	15	44	**1.0**	0.38
	No correl	4172	134	56	**2.6**	0.41
Alphaproteobacteria	Positive	3324	9	33	**0.3**	0.28
	No correl	2007	70	67	**2.2**	0.37
Gammaproteobacteria	Positive	48	2	0	**0.01**	**0.01**
	No correl	473	43	0	**1.7**	**0.58**
Flavobacteria	Positive	2377	16	33	**0.5**	**0.12**
	No correl	2126	49	67	**1.8**	**0.50**
Sphingobacteria	Positive	1435	4	25	**0.1**	**0.03**
	No correl	1363	32	75	**1.6**	**0.70**

*For this analysis, we used the full (non-rarefied) OTU table. Values in bold indicate significantly different diversity (Shannon) or MPD values (p < 0.05) between the groups within a given class. Within Gammaproteobacteria, all taxa were classified as “moderately abundant” (< 0.1% of the pooled sequences)*.

### Different environmental preferences among OTUs within bacterial classes

**Figure 6:**
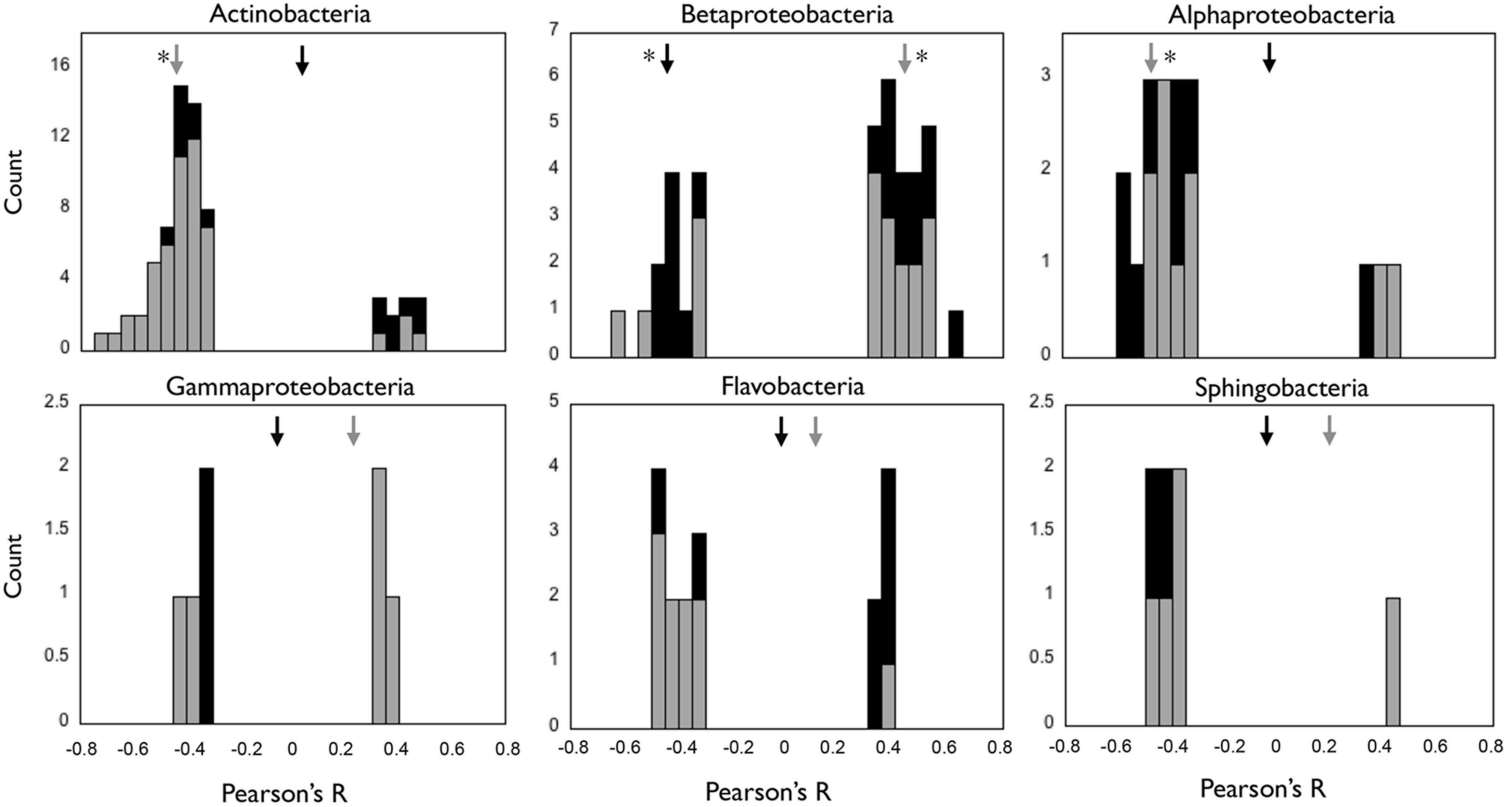
**Distribution of the Pearson's ***R*** values of the correlations between individual OTUs and environmental PCA axes PC1 (black bars) and PC2 (gray bars) for each of the six bacterial classes, and ***R*** values of the correlations at the class level (arrows)**. PC1 and PC2 depicted seasonal and spatial variation in environmental conditions, respectively (see Materials and Methods, Figure S4). Only those correlations that were significant (*p* < 0.05) are shown. Black and gray arrows indicate the *R* values of the correlation between the class abundance and PC1 and PC2 axes, respectively, and asterisks indicate those cases where these correlations were significant (*p* < 0.05).

We investigated the responses of the OTUs within each class to the environmental gradients generated by the reservoirs, and compared them to those observed for their harboring class. We run a PCA based on the environmental parameters (Figure S4), and explored correlations between the two first principal components and abundances of OTUs and classes (Figure 6). The PC axes summarized both the seasonal (winter vs. summer, PC1) and the spatial (upstream vs. downstream, PC2) differences in environmental conditions, and together explained 77% of total environmental variability. Interestingly, we found OTUs responding positively and negatively to the same PC axis within all classes (Figure 6), indicating the presence of taxa showing contrasting spatial and temporal behaviors within each class. Only Actinobacteria, Betaproteobacteria, and Alphaproteobacterial abundances showed significant correlations with the PC axes, and the patterns observed at the class level did not always accurately represent the ecological dynamics of their constituent OTUs. For example, in the case of Actinobacteria, the negative correlation observed at the class level with axis PC2 seemed to reflect the dynamics of a large group of OTUs that covaried negatively with PC2, but the negative correlation with PC1 observed for Betaproteobacteria did not capture the behavior of a group of OTUs that showed positive correlations with that same axis (Figure 6).

## Discussion

Despite some recent efforts to use trait-based approaches for understanding the ecology of bacterial groups (e.g., Lennon et al., 2012; Barberán et al., 2014; Fierer et al., 2014), attempts to assess the ecological coherence at high bacterial ranks are often based on relating abundance and distribution patterns of bacterial groups to environmental or spatio-temporal gradients. However, few studies have tested how uniformly these ecological patterns are shared among the members of a given lineage, which largely limits our ability to understand to which extent, or in which cases, these high-rank taxonomic categories can be regarded as ecologically meaningful units. By comparing the responses at the OTU and class level over an abrupt change in physicochemical conditions, we show that lineages that could have been regarded as ecologically meaningful groups based on their evident environmentally-driven abundance patterns (Ruiz-González et al., 2013a), do not always reflect the dynamics of all the dominant OTUs within them and actually comprise subsets of taxa displaying opposite responses to the same environmental gradients.

In accordance to our previous results (Ruiz-González et al., 2013a), here we show that the change in environmental conditions associated to the reservoirs promoted the development of different bacterial assemblages between the upstream and downstream reaches in the Ebro river, and shifts in abundance were observed for all bacterial classes passing through the reservoirs. Even though we did not attempt to provide absolute values for the presence of different taxa, we found remarkably similar trends between sequence- and CARD-FISH-based relative abundances, supporting that sequence patterns mirror the abundance dynamics at the class level (Figure S2). These changes in abundance, which were also strongly associated to variations in factors such as water temperature, conductivity, and dissolved inorganic nitrogen (Ruiz-González et al., 2013a), could lead to the conclusion that most taxa within these major taxonomic groups share some ecological traits (e.g., Fierer et al., 2007; Philippot et al., 2010). In most cases, though, these abundance shifts were associated to changes in alpha- and beta-diversity (Figure 2, Table 1), suggesting that not all members belonging to a same lineage responded similarly to changing conditions, and thus discarding a strong ecological coherence of bacteria at the class level.

It could be argued that such large shifts in intra-class composition may be due to continuous inputs of new taxa coming from different adjacent systems (e.g., tributaries, recruitment from soils or sediments, e.g., Crump et al., 2012; Jackson et al., 2014; Savio et al., 2015), in which case deductions about the ecological coherence of bacterial groups would be hampered by the blurring of possible ecological responses by mass effects (e.g., Nelson et al., 2009; Lindström and Langenheder, 2012; Ruiz-González et al., 2015a; Niño-García et al., in press). Our results discard this possibility by showing that in all bacterial classes, the vast majority of the sequences of reservoir and downstream bacterial assemblages belonged to taxa that were already present in upstream waters (92–99% of total sequences in both cases, Figure 3A). This implies that downstream communities comprised mostly bacteria imported from upstream waters, and thus, that the observed compositional shifts must reflect the selection of particular OTUs from the pool of taxa transported down the river. Indeed, when the taxa were categorized depending on the river reach where they were more prevalent, we observed remarkable shifts in their contribution to upstream, downstream, and reservoir populations within each class (Figure 3B), supporting that changes in the abundance of ubiquitous taxa were responsible for the observed shifts in taxonomic structure along the river. This is in accordance to previous studies that have shown that hydrologically driven dispersal followed by species sorting (i.e., selection by local physicochemical conditions, predation, viral lysis, competition with local communities) can largely determine the composition of downstream assemblages in freshwater ecosystems, even unveiling selection for particular bacterial classes or phyla (Lindström and Bergström, 2004; Crump et al., 2007, 2012; Read et al., 2015; Ruiz-González et al., 2015a; Savio et al., 2015; Niño-García et al., in press). Interestingly, the fact that the different classes assessed here displayed different compositional adjustments to the same change in environmental conditions (Figure 3) further suggests that the dominant process controlling the assembly of downstream populations also differ between classes.

The movement of water further determines the time frame that bacteria have to grow in response to local conditions (Crump et al., 2004, 2007; Lindström et al., 2006; Nelson et al., 2009; Read et al., 2015; Ruiz-González et al., 2015a; Niño-García et al., in press), and thus, besides the changes in environmental conditions, increases in water residence time within the impoundments may had favored the development of more lacustrine-like communities (e.g., Mašín et al., 2003). Since reservoir and downstream communities often presented similar overall composition, it could thus be argued that downstream assemblages are largely structured by mass immigration of bacteria from the reservoirs (Figures 1, 3). However, a detailed exploration of the OTU dynamics within each class showed compositionally different patterns between reservoir and downstream waters (details not shown), suggesting that bacteria do face new environmental or hydrological conditions beyond the reservoirs that force some of the taxa to a new adaptation.

We then investigated which fraction of the taxa within a class was represented by the dynamics of the whole class (Figure 4), and observed that only a small proportion of OTUs (4–25%) displayed positive correlations with class abundance. Since the phylogenetic diversity comprised by the studied classes is known to differ (e.g., Newton et al., 2011), we expected to find variable degrees of ecological coherence within them, such that some groups would provide a better representation of the ecological preferences of their constituent subtaxa than others, as reported elsewhere (Koeppel and Wu, 2012). For example, we expected that in highly abundant and diverse classes such as Actinobacteria, the class-level abundance patterns would fail to capture the dynamics of a larger fraction of its constituent OTUs than in smaller and less diverse classes, since even populations within Actinobacterial lineages have been shown to differ in their potential to adapt to different conditions (e.g., Ghylin et al., 2014). However, regardless of the large variations in sequence number, OTU richness and taxonomic diversity among classes (Figure S3), we did not observe large differences in the proportion of OTUs (and their associated sequences) positively correlating with the abundance at the class level: With the exception of Gammaproteobacteria, for which the small number of sequences recovered may preclude deriving strong conclusions, the fraction of OTUs behaving like their harboring class was relatively similar among bacterial classes (Figure 4). The fact that even in a single hydrologically connected system the dynamics of a large share of the members within each class (30–50% of the total sequences associated to each class) could not be inferred from the abundance patterns at the class level suggests that, if assessed over broader spatial or environmental scales, the potential ecological meaning of high-rank abundance patterns may be even smaller.

Interestingly, while in the three Proteobacterial classes the two sub-groups of OTUs (positively- and non-correlated OTUs) were associated to different taxonomic orders, this was not the case for the rest of the classes (Figure 5). This suggests that the phylogenetic depth at which bacteria group into ecologically coherent clusters does vary among different high-rank groups, in agreement with previous studies (Koeppel and Wu, 2012; Placella et al., 2012). In this sense, the ecological response of OTUs from the same taxonomic order seemed to be more conserved within Proteobacteria (mostly Alpha- and Gammaproteobacteria) than for the rest of the classes. Thus, aggregating bacterial diversity at the order rather than at the class level may be a better approach when looking for coherent ecological behaviors within Proteobacteria than for the rest of classes, for which this coherence would likely be more evident at lower taxonomic levels.

In general, the fraction of taxa within the subgroups of OTUs covarying with their corresponding class was less taxonomically and phylogenetically diverse than the uncorrelated taxa (Figure 5, Table 2). This is in accordance with the hypothesis that higher phylogenetic relatedness (i.e., lower MPD) reflects a large role of environmental filtering in community assembly, by assuming that closely related species are ecologically more similar (Webb et al., 2002; Horner-Devine and Bohannan, 2006), and therefore taxa responding similarly to changes in environmental conditions should have lower MPD among each other. Interestingly, this was also supported by the observation that the % of sequences associated to OTUs positively correlating with the class (Figure 4) seemed to increase with decreasing MPD at the class level (Figure S3), although this trend was only detectable when the class Gammaproteobacteria was excluded from the analysis (details not shown).

Had the OTUs not represented by the class's dynamics comprised only rare and non-responsive taxa, the abundance patterns at the class level would provide good insight into the ecological preferences of its dominant members. However, the fact that the non-correlated OTUs actually harbored a larger proportion of the “dominant” taxa within each class (Table 2), and that most classes comprised sub-groups of taxa showing opposite patterns along the environmental PC axes (i.e., contrasting ecological preferences, Figure 6), suggests that patterns observed at the class level may not even capture the dominant ecological responses of their constituent OTUs. Not surprisingly, the literature abounds in examples of lack of coupling between patterns in microbial taxonomic composition and function (Langenheder et al., 2005; Lear et al., 2014; Ruiz-González et al., 2015b). The question then becomes what affordable level of aggregation or definition we need for capturing true ecological strategies from amplicon-based information. Some lines of research are searching alternatives for the use of OTUs, such as those proposing the delineation of ecotypes as units for bacterial diversity (Ward et al., 2006; Koeppel and Wu, 2013), or those working on the development of clustering methods that do not rely in arbitrary thresholds (Mahé et al., 2014). Alternatively, the exploration of life strategies through clustering relative abundance patterns of bacterial OTUs seems to effectively capture ecological responses that cannot always be predicted from taxonomic patterns (Evans and Wallenstein, 2014; Shade et al., 2014; El-Swais et al., 2015; Ruiz-González et al., 2015a). Future studies in any of these directions will certainly provide insight into the significance of bacterial responses that may transcend phylogenetic categories, a necessary step toward the organization of bacterial diversity into a treatable number of ecologically meaningful units.

In summary, we show that, even in a single hydrologically connected system, a significant proportion of OTUs within dominant bacterial classes did not share the ecological trends observed for the class, including some of the dominant taxa. The presence of different subgroups of taxa within each class showing contrasting responses to the riverine environmental gradient calls for caution when the ecological attributes of high bacterial ranks are to be interpreted, and further highlights the need to elucidate ecologically coherent lower rank sub-clusters within bacteria for a more accurate understanding of the functioning of bacterial assemblages and their potential responses to changing environmental conditions.

### Conflict of interest statement

The authors declare that the research was conducted in the absence of any commercial or financial relationships that could be construed as a potential conflict of interest.
