# Supplementary material for: Weak Coherence in Abundance Patterns Between Bacterial Classes and Their Constituent OTUs Along a Regulated River
Source: Front Microbiol. 2015 Nov 26;6:1293. doi: 10.3389/fmicb.2015.01293 (PMC4659902; doi:10.3389/fmicb.2015.01293)

Fig. S1. OTU accumulation curve of all the sampled bacterioplankton communities, pooling the three samplings together (n=34, see Methods).

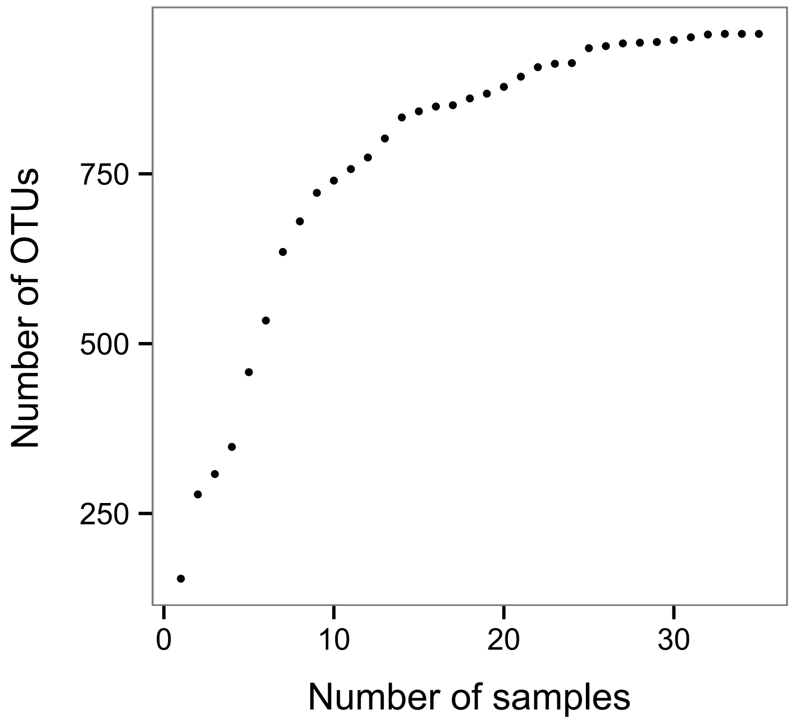

Supplement: Supplementary file 1 [file Image1.PDF]
