# Supplementary material for: Weak Coherence in Abundance Patterns Between Bacterial Classes and Their Constituent OTUs Along a Regulated River
Source: Front Microbiol. 2015 Nov 26;6:1293. doi: 10.3389/fmicb.2015.01293 (PMC4659902; doi:10.3389/fmicb.2015.01293)

Fig. S2. Comparison between the relative abundances of the different bacterial groups in terms of percentage of total reads (454 pyrosequencing data) and CARD-FISH counts under the microscope (published in Ruiz-González *et al*. 2013). All correlations are significant ($p<0.0001$) except for Gammaproteobacteria. Since we did not used specific probes for the Flavobacteria and Sphingobacteria classes within Bacteroidetes, the total number of sequences associated to the phylum Bacteroidetes were considered. Discrepancies in the percentages between both techniques may be due to the fact that while CARD-FISH targeted the whole bacterial assemblage, pyrosequencing was performed on the free-living bacterial community.

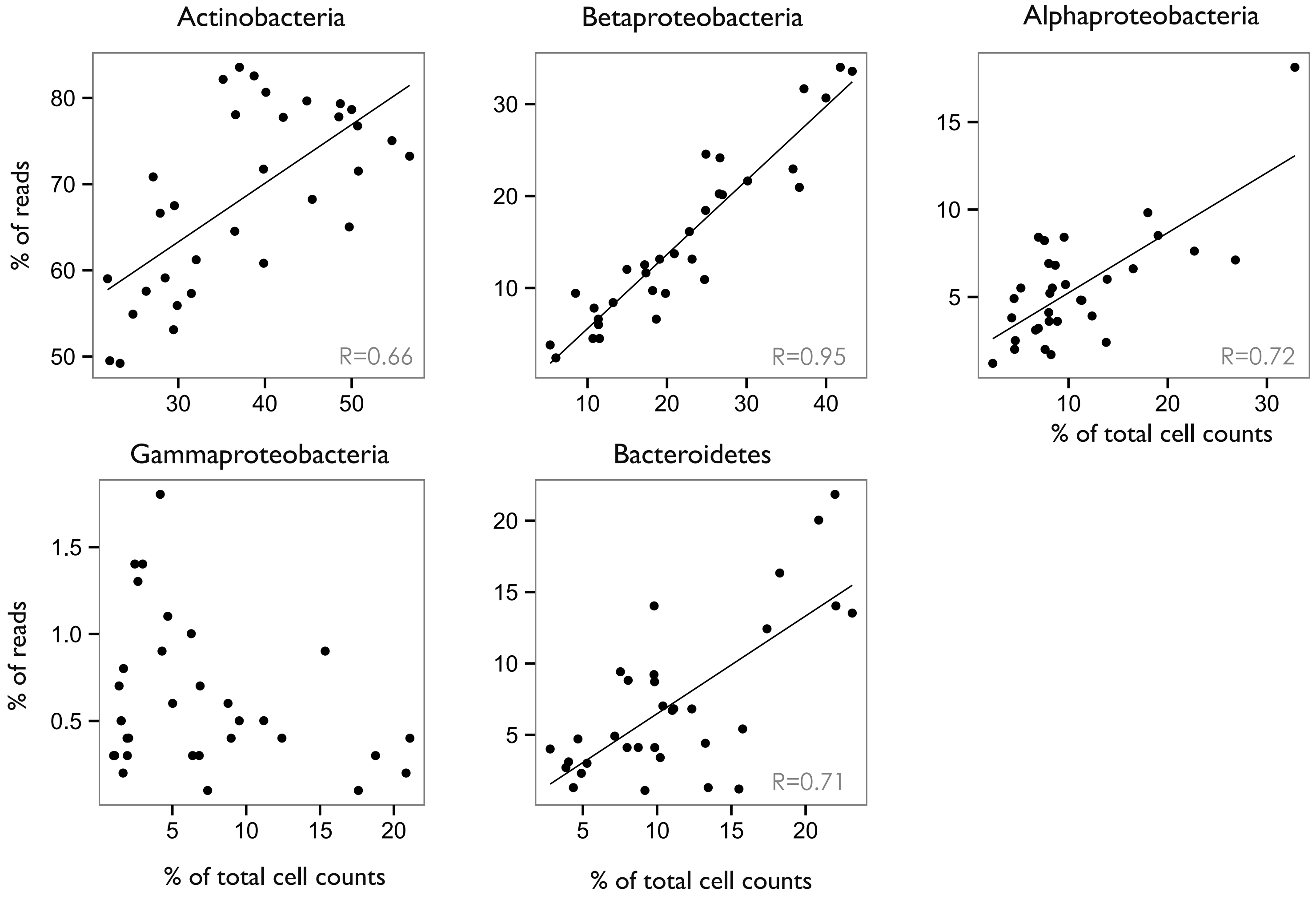

Supplement: Supplementary file 2 [file Image2.PDF]
