# Supplementary material for: Weak Coherence in Abundance Patterns Between Bacterial Classes and Their Constituent OTUs Along a Regulated River
Source: Front Microbiol. 2015 Nov 26;6:1293. doi: 10.3389/fmicb.2015.01293 (PMC4659902; doi:10.3389/fmicb.2015.01293)

Fig. S3. Changes in (A) sequence number, (B) OTU richness, (C) taxonomic diversity, and (D) mean pairwise distance among the six most abundant bacterial classes. Values are averages of all sampling sites over the 3 sampling periods, and were calculated from the rarefied OTU table (1000 sequences/sample, see Methods). [Actino] Actinobacteria; [Beta] Betaproteobacteria; [Alpha] Alphaproteobacteria; [Gamma] Gammaproteobacteria; [Flavo] C. Flavobacteria; [Sphing] Sphingobacteria.

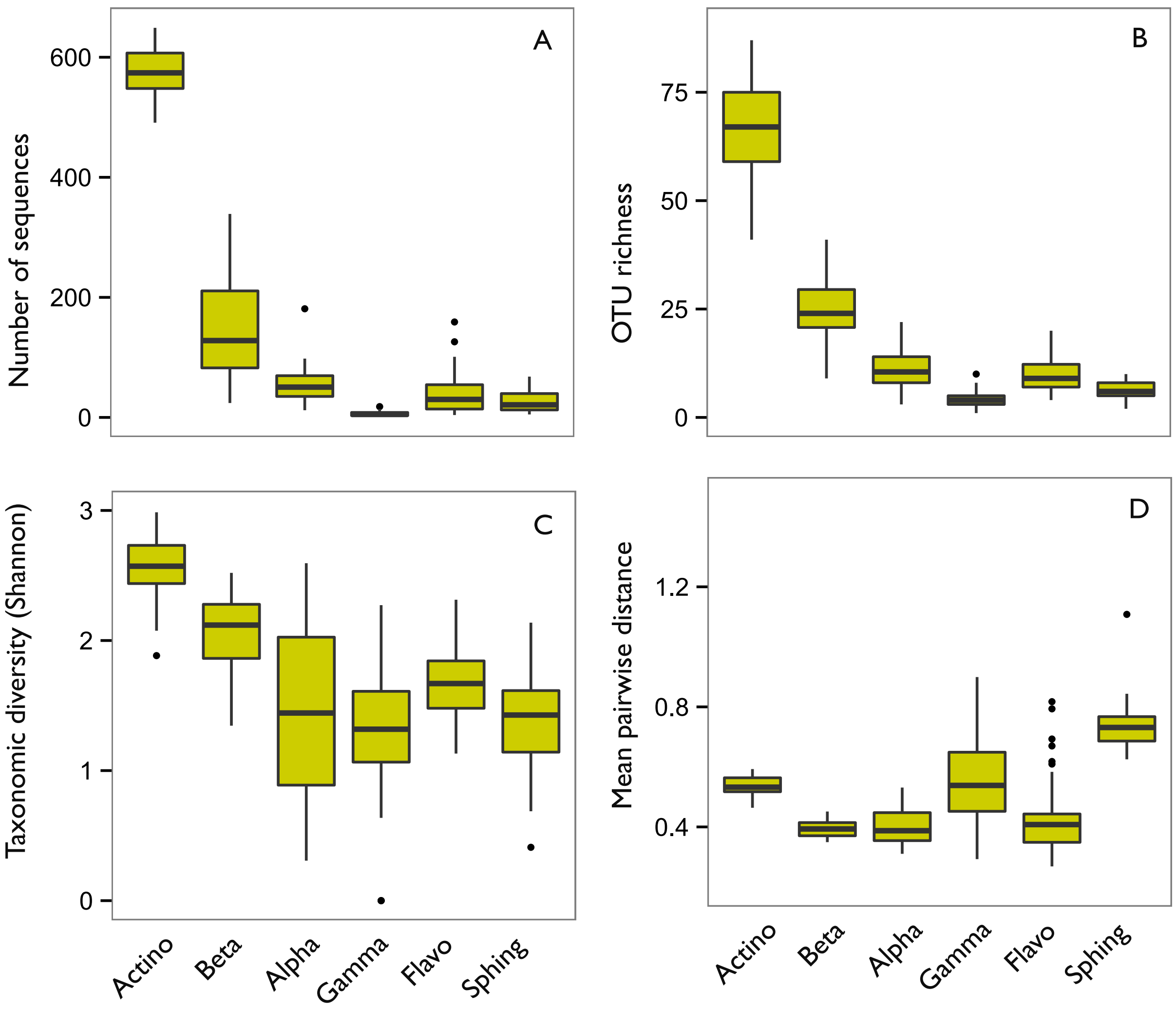

Supplement: Supplementary file 3 [file Image3.PDF]
