# Supplementary material for: Weak Coherence in Abundance Patterns Between Bacterial Classes and Their Constituent OTUs Along a Regulated River
Source: Front Microbiol. 2015 Nov 26;6:1293. doi: 10.3389/fmicb.2015.01293 (PMC4659902; doi:10.3389/fmicb.2015.01293)

Fig. S4. (A) Principal component analysis (PCA) of samples based on the measured environmental parameters. Colors indicate the different river reaches, and shape indicate the three sampling campaigns: July (squares), September (circles), and December (triangles). [DIN, NO3] Dissolved inorganic nitrogen and nitrate concentration; [Seston] ;[Cond] Conductivity; [Chl*a*] Chlorophyll *a* concentration; [Phyto] Phytoplankton abundance; [TDP, DOP] Total dissolved phosphorus and dissolved organic phosphorus concentration; [TDC] Total dissolved carbon concentration; [Temp] Temperature.

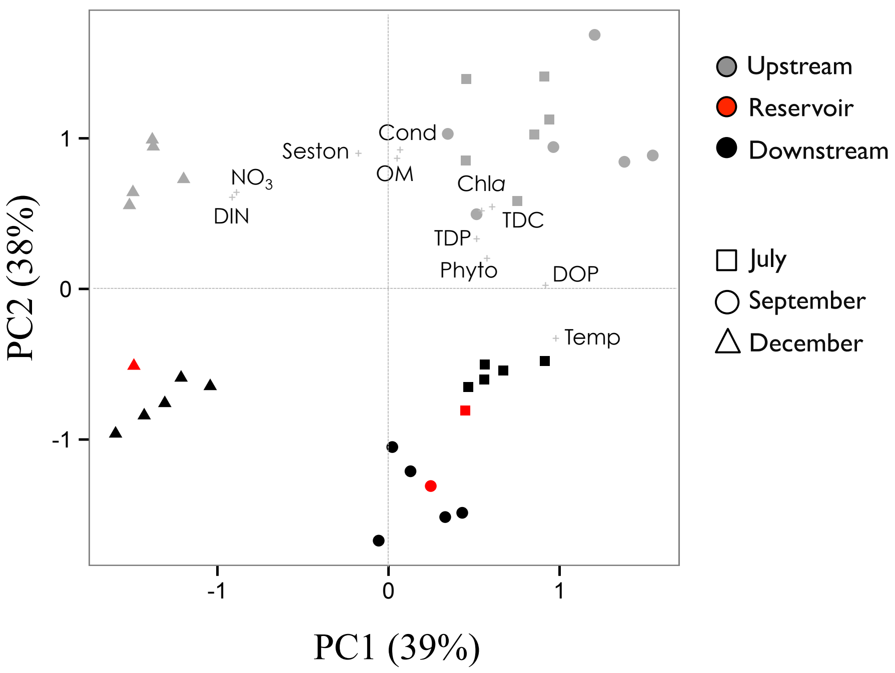

Supplement: Supplementary file 4 [file Image4.PDF]
